# Bioactive Eunicellin-Based Diterpenoids from the Soft Coral *Cladiella krempfi*

**DOI:** 10.3390/md9102036

**Published:** 2011-10-19

**Authors:** Chi-Jen Tai, Jui-Hsin Su, Ming-Shyan Huang, Zhi-Hong Wen, Chang-Feng Dai, Jyh-Horng Sheu

**Affiliations:** 1 Department of Marine Biotechnology and Resources, National Sun Yat-sen University, Kaohsiung 804, Taiwan; E-Mails: jean801023@hotmail.com (C.-J.T.); wzh@mail.nsysu.edu.tw (Z.-H.W.); 2 National Museum of Marine Biology & Aquarium, Pingtung 944, Taiwan; E-Mail: x2219@nmmba.gov.tw; 3 Graduate Institute of Marine Biotechnology, National Dong Hwa University, Pingtung 944, Taiwan; 4 Department of Internal Medicine, Kaohsiung Medical University, Kaohsiung 807, Taiwan; E-Mail: shyang@cc.kmu.edu.tw; 5 Chung-Ho Memorial Hospital, Kaohsiung Medical University, Kaohsiung 807, Taiwan; 6 Institute of Oceanography, National Taiwan University, Taipei 112, Taiwan; E-Mail: corallab@ntu.edu.tw; 7 Division of Marine Biotechnology, Asia-Pacific Ocean Research Center, National Sun Yat-sen University, Kaohsiung 804, Taiwan

**Keywords:** soft coral, *Cladiella krempfi*, eunicellin-based diterpenoids, krempfielins, cytotoxic activity, anti-inflammatory activity

## Abstract

Four new eunicellin-based diterpenoids, krempfielins A–D (**1**–**4**), along with two known compounds (**5** and **6**) have been isolated from a soft coral *Cladiella krempfi*. The structures of the new metabolites were elucidated by extensive spectroscopic analysis and by comparison with spectroscopic data of related known compounds. Compounds **5** and **6** were shown to exhibit cytotoxicity against a limited panel of cancer cell lines. Furthermore, compounds **2**, **3**, **5** and **6** were shown to exert significant *in vitro* anti-inflammatory activity against LPS-stimulated RAW264.7 macrophage cells.

## 1. Introduction

**Chart 1:**
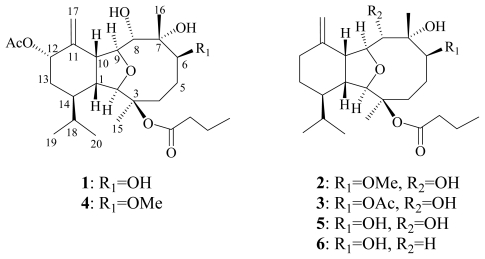
Structures of metabolites **1**–**6**.

In previous studies, a series of novel secondary metabolites, including one eunicellin-based diterpenoid [1] and pregnane-type steroids have been isolated from the soft coral *Cladiella krempfi* [2–4]. During the course of our search for bioactive metabolites from marine invertebrates of Taiwanese waters, several eunicellin-type compounds also have been isolated from octocorals *Pachyclavularia violacea* [5,6], *Cladiella australis* [7], *Cladiella hirsuta* [8], *Vigularia juncea* [9], *Klyxum simplex* [10–14]. A related study from an Indonesian soft coral *Cladiella* sp. also afforded diterpenes of this type [15]. Recently, our investigation on the chemical constituents of the Formosan soft coral *Cladiella krempfi* yielded four new eunicellin-type metabolites, krempfielins A–D (**1**–**4**), along with two known eunicellin-based diterpenoids, litophynol B (**5**) [16] and (1R*, 2R*, 3R*, 6S*, 7S*, 9R*, 10R*, 14R*)-3-butanoyloxycladiell-11(17)-en-6,7-diol (**6**) [17] (Chart 1). These compounds possess the more common C-2–C-9 ether linkage characteristic of the eunicellin-based diterpenoids. The molecular structures of these compounds, including their relative stereochemistries, were established by the detailed spectroscopic analysis and by comparison with related physical and spectral data from known compounds. The cytotoxicity of compounds **1**–**6** against five human tumor cell lines, lung adenocarcinoma (A549 and H1299), breast carcinoma (BT483), liver carcinoma (HepG2), oral cancer (SAS) and one human lung bronchial cell line (BEAS2B) was evaluated. The ability of **1**–**6** to inhibit the up-regulation of pro-inflammatory iNOS (inducible nitric oxide synthase) and COX-2 (cyclooxygenase-2) proteins in LPS (lipopolysaccharide)-stimulated RAW264.7 macrophage cells was also evaluated.

## 2. Results and Discussion

**Figure 1:**
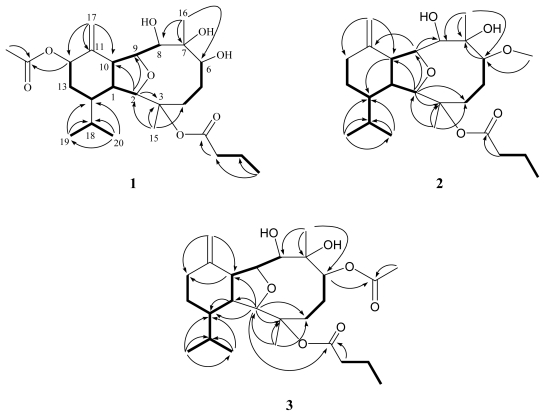
Selected ^1^H–^1^H COSY (**—**) and HMBC (→) correlations of **1**–**3**.

Krempfielin A (**1**) was obtained as a colorless oil. The HRESIMS (*m*/*z* 505.2777 [M + Na]^+^) of **1** established a molecular formula of C_26_H_42_O_8_, implying six degrees of unsaturation. The IR spectrum of **1** revealed the presence of hydroxyl and carbonyl groups from absorptions at 3445 and 1730 cm^−1^, respectively. The ^13^C NMR spectroscopic data of **1** exhibited 26 carbon signals (Table 1), which were assigned by the assistance of DEPT spectrum to six methyls (including one acetate methyl at *δ*_C_ 21.9), six methylenes (including one sp^2^ methylene at *δ*_C_ 114.9), nine methines (including five oxymethines at *δ*_C_ 90.5, 81.9, 78.8, 77.0 and 72.9), five quaternary carbons (including two sp^2^ oxygenated quaternary carbons at *δ*_C_ 172.3 and 170.5, two sp^3^ oxygenated quaternary carbons at *δ*_C_ 86.0 and 79.3, and one sp^2^ quaternary carbon at *δ*_C_ 143.7). The NMR data of **1** (Tables 1 and 2) showed the appearance of a terminal methylene group (*δ*_C_ 114.9, CH_2_ and 143.7, qC; *δ*_H_ 5.20 brs), a isopropyl moiety (*δ*_C_ 28.9, CH; 21.5, CH_3_; and 16.6, CH_3_ and *δ*_H_ 1.85, m, 1H; 0.97, d, 3H, *J* = 6.6 Hz and 0.86, d, 3H, *J* = 6.6 Hz), one *n*-butyrate (*δ*_C_ 172.3, qC; 37.3, CH_2_; 18.3, CH_2_ and 13.6, CH_3_; and *δ*_H_ 2.28, m, 2H; 1.65, m, 2H and 0.97, t, 3H, *J* = 7.2 Hz) and one acetate group (*δ*_C_ 170.5, qC and 21.9, CH_3_ and *δ*_H_ 2.09, s, 3H), respectively. Analysis of HMQC correlations showed that proton signals appearing at *δ*_H_ 2.34 (1H, m), 3.30 (1H, t, *J* = 5.7 Hz), 3.69 (1H, s), and 4.05 (1H, dd, *J* = 9.3, 5.7 Hz) were correlated to two ring juncture methine carbons at *δ*_C_ 43.6 and 50.3 and two oxymethine carbons at *δ*_C_ 90.5 and 81.9, respectively. This suggested the presence of a tetrahydrofuran structural unit. In addition, the ^1^H–^1^H COSY correlations of **1** assigned three isolated consecutive proton spin systems (Figure 1). The molecular framework of **1** was further established by HMBC data (Figure 1). Furthermore, H-12 (*δ* 5.44) and an acetate methyl exhibited HMBC correlations to the acetate carbonyl carbon (*δ* 170.5), revealing the location of an acetate at C-12. The location of a *n*-butyrate at C-3 was then deduced by the chemical shifts of C-3 (*δ* 86.0) and H_3_-15 (*δ* 1.47). From the above results, the structure of compound **1** was shown to be highly related to that of a known compound, litophynol B (**5**) [16].

**Figure 2:**
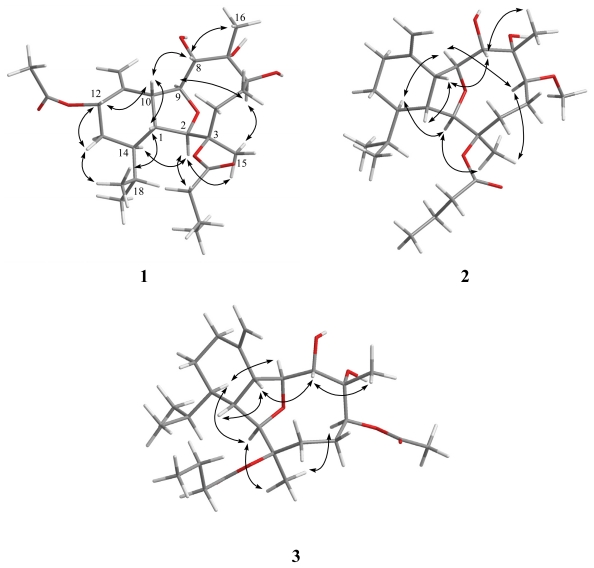
Key NOESY correlations for **1**–**3**.

The relative configuration of **1** was mostly confirmed to be the same as that of **5** by comparison of the chemical shifts of both compounds and was further confirmed by NOE correlations (Figure 2). Furthermore, one additional NOE correlation between H-10 with H-12 suggested that H-12 was β-oriented and the relative configuration of **1** was proposed as 1R*, 2R*, 3R*, 6S*, 7R*, 8S*, 9S*, 10R*, 12S*, and 14R*.

The HRESIMS of krempfielin B (**2**) exhibited a [M + Na]^+^ peak at *m*/*z* 461.2881 and established a molecular formula of C_25_H_42_O_6_, appropriate with five degrees of unsaturation. By comparison of the ^1^H and ^13^C NMR data of **2** with those of **5**, it was found that they were very similar. However, a methoxyl group (*δ*_H_ 3.36, 3H, s; *δ*_C_ 56.95, CH_3_) was observed in **2**. In addition, the position of methoxyl group at C-6 was confirmed by the HMBC correlation of the methoxyl proton (*δ*_H_ 3.36) to an oxymethine carbon (*δ*_C_ 88.3, CH, C-6). A more detailed analysis of the ^1^H and ^13^C NMR spectroscopic data and correlations in the ^1^H–^1^H COSY and HMBC spectra led to the establishment of the gross structure of **2** (Figure 1). The NOESY correlations of **2** (Figure 2) also showed the stereochemistry similarity between compounds **2** and **5**. All of the above information suggested that **2** was the 6-*O*-methyl derivative of **5**.

The molecular formula C_26_H_42_O_7_ with six degrees of unsaturation was assigned to krempfielin C (**3**) from its HRESIMS data (*m*/*z* 489.2829 [M + Na]^+^). The NMR spectroscopic data of **3** (Tables 1 and 2) showed the presence of one acetoxy group (*δ*_C_ 171.9, qC and 21.4, CH_3_; and *δ*_H_ 2.09 s, 3H) and one *n*-butyryloxy group (*δ*_C_ 172.4, qC; 37.4, CH_2_; 18.4, CH_2_ and 13.6, CH_3_; and *δ*_H_ 2.34 m, 1H; 2.31 m, 1H; 1.67 m, 2H and 0.99 t, 3H, *J* = 7.2 Hz). NMR data of **3** showed similarities with those of **5**, except for the presence of an acetoxyl group at C-6 of **3** that downfielded H-6 to *δ*_H_ 5.72 and C-6 to *δ*_C_ 82.2 ppm. These observations could be further confirmed by the correlations observed in the 2D NMR (including ^1^H–^1^H COSY, HMBC and NOESY) experiments of **3** (Figures 1 and 2).

Krempfielin D (**4**) was isolated as a colorless oil with a molecular formula C_27_H_44_O_8_ which possesses six units of unsaturation, as indicated by HRESIMS (*m*/*z* 519.2934). The ^1^H and ^13^C NMR spectral data of **4** (Tables 1 and 2) revealed that the structure of metabolite **4** should be similar to that of **1**, as the NMR spectral data of **4** are almost identical with those of **1** except for the presence of a methoxyl group (*δ*_H_ 3.36, 3H, s) in **4**. Also, the ^13^C NMR spectrum of **4** showed the same number of methylene, methine, and quaternary carbons as that of **1**, except for the presence of a methoxyl carbon, which showed a signal at *δ*_C_ 56.8 (qC). Furthermore, the methoxyl protons gave an HMBC cross-peak with an oxymethine carbon (*δ* 87.4, CH), indicating the presence of the methoxyl group at C-6 in **4**. The stereochemistry of **4** was confirmed by comparison of the NMR data and NOE correlations of both **1** and **4**.

**Figure 3:**
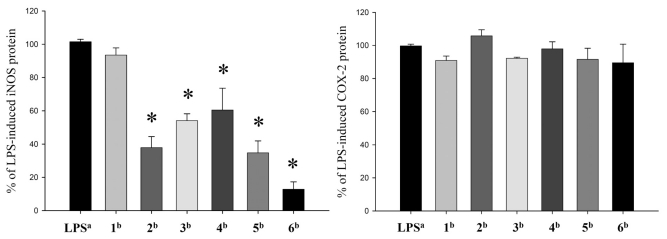
Effect of compounds **1**–**6** on lipopolysaccharide (LPS)-induced inducible nitric oxide synthetase (iNOS) and cyclooxygenase-2 (COX-2) proteins expression in RAW264.7 macrophage cells by immunoblot analysis. The values are mean ± SEM. (*n* = 6). Relative intensity of the LPS alone stimulated group was taken as 100%. * Significantly different from LPS alone stimulated group (* *P* < 0.05). ^a^ stimulated with LPS; ^b^ stimulated with LPS in the presence of **1**–**6** (10 μM).

The cytotoxicity of the diterpenoids **1**–**6** against five human carcinoma cell lines A549, H1299, BT483, HepG2, SAS and one human normal cell line BEAS2B was evaluated by the MTT assay. It was found that only **5** showed activity against the proliferation of H1299 and BT483 cancer cells (ED_50_ values of 18.1 ± 1.5, and 13.2 ± 1.1 μg/mL), and **6** exhibited cytotoxicity toward A549, BT483 and SAS cancer cell lines (ED_50_ values of 15.8 ± 2.0, 8.5 ± 1.0 and 14.3 ± 1.8 μg/mL), respectively. Furthermore, **5** and **6** were found to be non-cytotoxic toward the normal cell BEAS2B. In the present study, the *in vitro* anti-inflammatory effects of compounds **1**–**6** were also tested by examining the inhibitory activity of these compounds toward the LPS-induced up-regulation of pro-inflammatory proteins, iNOS and COX-2 in RAW264.7 macrophage cells (Figure 3). At a concentration of 10 μM, compounds **2**–**6** were found to significantly reduce the levels of iNOS protein, relative to the control cells stimulated with LPS only. However, these metabolites did not effectively reduce the expression of COX-2 protein.

## 3. Experimental Section

### 3.1. General Experimental Procedures

Optical rotations were measured on a JASCO P-1020 polarimeter. IR spectra were recorded on a JASCO FT/IR-4100 infrared spectrophotometer. ESIMS and HRESIMS were obtained with a Bruker APEX II mass spectrometer. The NMR spectra were recorded in CDCl_3_ either on a Varian UNITY INOVA-500 FT-NMR, a Varian 400MR FT-NMR or a Bruker AMX-300 FT-NMR. Silica gel (Merck, 230–400 mesh) was used for column chromatography. Precoated silica gel plates (Merck, Kieselgel 60 F-254, 0.2 mm) were used for analytical TLC. High performance liquid chromatography was performed on a Hitachi L-7100 HPLC apparatus with an ODS column (250 × 21.2 mm, 5 mm).

### 3.2. Animal Material

*C. krempfi* was collected by hand using scuba off the coast of Penghu islands of Taiwan in June 2008, at a depth of 5–10 m, and stored in a freezer until extraction. A voucher sample was deposited at the Department of Marine Biotechnology and Resources, National Sun Yat-sen University.

### 3.3. Extraction and Separation

The octocoral (1.1 kg fresh wt) was collected and freeze-dried. The freeze-dried material was minced and extracted exhaustively with EtOH (3 × 10 L). The EtOH extract of the frozen organism was partitioned between CH_2_Cl_2_ and H_2_O. The CH_2_Cl_2_-soluble portion (14.4 g) was subjected to column chromatography on silica gel and eluted with EtOAc in *n*-hexane (0–100% of EtOAc, stepwise) and then further with MeOH in EtOAc with increasing polarity to yield 41 fractions. Fraction 28, eluted with *n*-hexane–EtOAc (1:2), was rechromatoraphed over a RP-18 open column using acetone–H_2_O (10:1) as the mobile phase to afford six subfractions (A1–A6). Subfraction A1 was separated by reverse phase HPLC (CH_3_CN–H_2_O, 1:1 to 2:1) to afford compounds **1** (8.3 mg), **2** (3.5 mg), **3** (6.2 mg), **5** (12.2 mg) and **6** (21.3 mg). Subfraction A2 separated by reverse phase HPLC (CH_3_CN–H_2_O, 3.8:1) to afford compound **4** (5.0 mg).

Krempfielin A (**1**): colorless oil; [α]_D_ ^25^ −39.2 (*c* 0.83, CHCl_3_); IR (neat) ν_max_ 3445, 2919, 1730, 1648, 1462, 1375, 1243, 1183 and 1043 cm^−1; 1^H and ^13^C NMR data, see Tables 1 and 2; ESIMS *m/z* 505 [M + Na]^+^; HRESIMS *m/z* 505.2775 [M + Na]^+^ (calcd for C_26_H_42_O_8_Na, 505.2777).

Krempfielin B (**2**): colorless oil; [α]_D_ ^25^ −62.9 (*c* 0.35, CHCl_3_); IR (neat) ν_max_ 3461, 2931, 1735, 1645, 1456, 1370, 1251, 1174 and 1046 cm^−1; 1^H and ^13^C NMR data, see Tables 1 and 2; ESIMS *m/z* 461 [M + Na]^+^; HRESIMS *m/z* 461.2881 [M + Na]^+^ (calcd for C_25_H_42_O_6_Na, 461.2879).

Krempfielin C (**3**): colorless oil; [α]_D_ ^25^ −51.3 (*c* 0.62, CHCl_3_); IR (neat) ν_max_ 3471, 2931, 1733, 1647, 1456, 1370, 1251, 1176 and 1081 cm^−1; 1^H and ^13^C NMR data, see Tables 1 and 2; ESIMS *m/z* 489 [M + Na]^+^; HRESIMS *m/z* 489.2829 [M + Na]^+^ (calcd for C_26_H_42_O_7_Na, 489.2828).

Krempfielin D (**4**): colorless oil; [α]_D_ ^25^ −52.4 (*c* 0.5, CHCl_3_); IR (neat) ν_max_ 3462, 2924, 1733, 1651, 1456, 1372, 1240, 1176 and 1080 cm^−1; 1^H and ^13^C NMR data, see Tables 1 and 2; ESIMS *m/z* 519 [M + Na]^+^; HRESIMS *m/z* 519.2934 [M + Na]^+^ (calcd for C_27_H_44_O_8_Na, 519.2937).

### 3.4. Cytotoxicity Testing

Cell lines were purchased from the American Type Culture Collection (ATCC). Cytotoxicity assays of compounds **1**–**6** were performed using the MTT [3-(4,5-dimethylthiazol-2-yl)-2,5-diphenyltetrazolium bromide] colorimetric method [18,19].

### 3.5. *In Vitro* Anti-Inflammatory Assay

Macrophage (RAW264.7) cell line was purchased from ATCC. *In vitro* anti-inflammatory activities of compounds **1**–**6** were measured by examining the inhibition of lipopolysaccharide (LPS) induced upregulation of iNOS (inducible nitric oxide synthetase) and COX-2 (cyclooxygenase-2) proteins in macrophages cells using western blotting analysis [20,21].

## Figures and Tables

**Table 1:** ^13^C NMR data for compounds **1**–**4**.

	1 a	2 a	3 b	4 c
1	43.6 (CH) d	45.5 (CH)	45.5 (CH)	43.9 (CH)
2	90.5 (CH)	92.3 (CH)	92.6 (CH)	92.6 (CH)
3	86.0 (qC)	86.1 (qC)	86.1 (qC)	85.9 (qC)
4	34.0 (CH_2_)	36.2 (CH_2_)	35.4 (CH_2_)	35.4 (CH_2_)
5	30.0 (CH_2_)	26.6 (CH_2_)	28.5 (CH_2_)	26.4 (CH_2_)
6	77.0 (CH)	88.3 (CH)	82.2 (CH)	87.4 (CH)
7	79.3 (qC)	78.7 (qC)	78.2 (qC)	78.4 (qC)
8	78.8 (CH)	79.6 (CH)	80.1 (CH)	79.1 (CH)
9	81.9 (CH)	81.7 (CH)	81.4 (CH)	82.5 (CH)
10	50.3 (CH)	53.2 (CH)	53.5 (CH)	50.7 (CH)
11	143.7 (qC)	148.4 (qC)	148.6 (qC)	143.2 (qC)
12	72.9 (CH)	31.7 (CH_2_)	31.5 (CH_2_)	73.1 (CH)
13	29.2 (CH_2_)	24.9 (CH_2_)	24.9 (CH_2_)	28.9 (CH_2_)
14	37.9 (CH)	44.2 (CH)	44.1 (CH)	37.6 (CH)
15	23.2 (CH_3_)	23.0 (CH_3_)	22.8 (CH_3_)	23.1 (CH_3_)
16	17.8 (CH_3_)	18.3 (CH_3_)	18.4 (CH_3_)	18.5 (CH_3_)
17	114.9 (CH_2_)	114.9 (CH_2_)	110.6 (CH_2_)	116.0 (CH_2_)
18	28.9 (CH)	29.0 (CH)	29.0 (CH)	28.8 (CH)
19	21.5 (CH_3_)	21.9 (CH_3_)	21.9 (CH_3_)	21.8 (CH_3_)
20	16.6 (CH_3_)	15.8 (CH_3_)	15.5 (CH_3_)	16.2 (CH_3_)
3-*n*-butyrate	172.3 (qC)	172.3 (qC)	172.4 (qC)	172.3 (qC)
	37.3 (CH_2_)	37.4 (CH_2_)	37.4 (CH_2_)	37.3 (CH_2_)
	18.3 (CH_2_)	18.3 (CH_2_)	18.4 (CH_2_)	18.3 (CH_2_)
	13.6 (CH_3_)	13.6 (CH_3_)	13.6 (CH_3_)	13.6 (CH_3_)
6-OMe		56.9 (CH_3_)		56.8 (CH_3_)
6-OAc			171.9 (qC)	
			21.4 (CH_3_)	
12-OAc	170.5 (qC)			170.3 (qC)
	21.9 (CH_3_)			21.6 (CH_3_)

a Spectra recorded at 75 MHz in CDCl_3_;

b Spectra recorded at 100 MHz in CDCl_3_;

c Spectra recorded at 125 MHz in CDCl_3_;

d Deduced from DEPT.

**Table 2:** ^1^H NMR data for compounds **1**–**4**.

	1 a	2 a	3 b	4 c
1	2.34 m	2.25 m	2.22 m	2.32 m
2	3.69 s	3.60 s	3.64 s	3.64 s
4	1.93 m; 2.43 m	1.76 m; 2.66 dd (14.4, 9.0)	1.97 m; 2.59 dd (15.2, 8.8)	1.84 m; 2.55 dd (14.0, 10.0)
5	1.50 m; 1.71 m	1.36 m; 1.63 m	1.46 m; 1.51 m	1.35 m; 1.68 m
6	4.63 d (7.2) d	4.13 d (6.3)	5.72 d (4.4)	4.12 d (8.5)
8	3.48 d (9.3)	3.54 t (9.3)	3.58 brt (9.2)	3.45 t (9.5)
9	4.05 dd (9.3, 5.7)	3.89 dd (9.3, 6.9)	3.84 dd (9.2, 7.2)	4.09 dd (9.5, 6.5)
10	3.30 t (5.7)	3.32 t (6.9)	3.36 t (7.2)	3.33 t (6.5)
12	5.44 d (3.3)	2.06 m; 2.32 m	2.05 m; 2.31 m	5.46 dd (5.0, 2.5)
13	1.50 m; 1.88 m	1.07 m; 1.75 m	1.06 m; 1.76 m	1.46 m; 1.90 m
14	1.68 m	1.29 m	1.25 m	1.67 m
15	1.47 s	1.41 s	1.38 s	1.46 s
16	1.26 s	1.24 s	1.29 s	1.23 s
17	5.20 brs	4.79 s, 4.90 s	4.79 s, 4.89 s	5.20 s, 5.21 s
18	1.85 m	1.72 m	1.69 m	1.82 m
19	0.97 d (6.6)	0.96 d (6.6)	0.97 d (6.8)	0.96 d (6.5)
20	0.86 d (6.6)	0.79 d (6.6)	0.79 d (6.8)	0.85 d (6.5)
3-*n*-butyrate	2.28 m	2.34 m	2.31 m, 2.34 m	2.17 m, 2.29 m
	1.65 m	1.67 m	1.67 m	1.59 m
	0.97 t (7.2)	0.99 t (7.5)	0.99 t (7.2)	0.97 t (7.5)
6-OMe		3.36 s		3.36 s
6-OAc			2.09 s	
12-OAc	2.09 s			2.08 s

a Spectra recorded at 300 MHz in CDCl_3_;

b Spectra recorded at 400 MHz in CDCl_3_;

c Spectra recorded at 500 MHz in CDCl_3_;

d *J* values (Hz) in parentheses.
